# Dawn of Precision Medicine in Psoriatic Arthritis

**DOI:** 10.3389/fmed.2022.851892

**Published:** 2022-03-18

**Authors:** Ippei Miyagawa, Yoshiya Tanaka

**Affiliations:** The First Department of Internal Medicine, University of Occupational and Environmental Health Japan, Kitakyushu, Japan

**Keywords:** psoriatic arthritis, precision medicine, immune cell phenotyping, targeted therapy, treatment

## Abstract

The establishment of precision medicine is considered particularly important in heterogeneous autoimmune diseases (e.g., psoriatic arthritis, systemic lupus erythematosus), which reveal clinical and molecular heterogeneity. The selection of optimal treatment strategies for individual patients may be more important and complex in autoimmune diseases than in other diseases. Two factors are important in precision medicine: patient stratification and use of targeted. When both factors work, patients are likely to have good outcomes. However, research into precision medicine and its practice in systemic autoimmune diseases is lacking. In contrast, the usefulness of peripheral immune cell phenotyping in the evaluation of immunological characteristics and stratification into subgroups of individual patients with systemic autoimmune diseases such as immunoglobulin 4-related disease, systemic lupus erythematosus, and anti-neutrophil cytoplasmic antibody-related vasculitis was reported. Furthermore, the potential of precision medicine using biological disease-modifying antirheumatic drugs based on peripheral immune cell phenotyping was recently demonstrated for psoriatic arthritis in the clinical setting. Precision medicine has not yet been sufficiently investigated in real world clinical settings. However, a dawn of precision medicine has emerged. We should shed further light on precision medicine in PsA and other autoimmune diseases. Here, we first review the usefulness of peripheral immune cell phenotyping in systemic autoimmune diseases and the potential of precision medicine in PsA based on this method.

## Introduction

In cancer care, genomic research has garnered attention since around 1990. Ever since the Precision Medicine Initiative for cancer care was proposed in the State of the Union address delivered by then-US President Obama in January 2015, attention has been drawn to precision medicine, wherein patients with cancer are classified based on oncogenic driver mutations, and the molecular-targeted drug that best matches each mutation is used for treatment (1).

The establishment of precision medicine is considered particularly important in heterogeneous autoimmune diseases such as psoriatic arthritis (PsA), systemic lupus erythematosus (SLE) and rheumatoid arthritis (RA). These diseases show clinical and molecular heterogeneity. In these diseases, various symptoms require simultaneous improvement; however, the number of available treatment options is limited. With the elucidation of pathological conditions of these autoimmune diseases, molecules involved in the pathological processes have been clarified. It has become clearer which molecules should now be targeted for treatment. Advances in monoclonal antibody technology, and the emergence of Janus kinase inhibitors (JAK-i) have made molecular-targeted therapy possible. However, in many autoimmune diseases, due to the heterogeneity of the disease, centrally involved molecules differ depending on individual patient. Differences in patient's molecular profiles can render molecular-targeted therapy inefficient. Therefore, patient stratification according to differences in the patient's molecular profile would allow for more efficient treatment outcomes by precision medicine as opposed to one-size-fits-all approach (2). However, although precision medicine is being developed for cancer and rare diseases, research on its development and use in systemic autoimmune and rheumatic diseases is lacking.

PsA is a rheumatic disease with high clinical heterogeneity; it is sometimes accompanied by nail psoriasis, spine, entheses, and eyes (iritis). In the pathogenesis of PsA, various cytokines such as interferon gamma, interleukin (IL)-12, IL-23, IL-17, IL-6, and tumor necrosis factor alpha (TNF-α) play important roles. Recently, biological disease-modifying antirheumatic drugs (bDMARDs) targeting TNF-α, IL-17A, IL-17A/F, IL-17 receptor, IL-12/23 (p40), and IL-23 (p19) and targeted synthetic DMARDs (tsDMARDs) targeting Janus kinase have demonstrated efficacy and are widely used in routine clinical practice (3–22). Abatacept, a selective T-cell co-stimulation modulator, has been also approved by US Food and Drug Administration (23). EULAR recommends using targeted therapies, such as TNF-inhibitors (TNF-i), IL-17-inhibitors (IL-17-i), IL-12/23-inhibitors (IL-12/23-i), JAK-i, and phosphodiesterase 4 inhibitors (PDE4-i), especially in patients with PsA who fail to adequately respond to synthetic DMARDs (24). Therefore, first of all, it is important to consider whether a patient needs to use biologics or not. Despite the availability of b/tsDMARDs, some patients are resistant to treatment. While these drugs target different molecules, clinical trials directly comparing TNF-i and IL-17-i have shown at least a comparable efficacy on musculoskeletal manifestations (25, 26). This suggests that individual patients diagnosed with PsA may possess different therapeutic targets to attain an optimal response. The diversity of pathologies in patients with such heterogeneous diseases may result in treatment resistance if key aspects or molecules of a disease in that individual are not properly targeted. However, no optimal drug selection method has been established, and some patients are resistant to these drugs and require treatment changes.

To date, precision medicine has not been achieved for any autoimmune disease. In contrast, we recently demonstrated the usefulness of peripheral immune cell phenotyping in the evaluation of immunological characteristics (phenotypic differences) and stratification of patients into subgroups in individual patients with IgG4-related disease (IgG4-RD), SLE, RA, and anti-neutrophil cytoplasmic antibody (ANCA)-related vasculitis (27–30). Moreover, we reported the potential of precision medicine based on this method in the real clinical setting of PsA (31). Here, we first review the usefulness of peripheral immune cell phenotyping in some systemic autoimmune diseases and the potential of precision medicine in PsA based on this method.

## Application of Peripheral Immune Cell Phenotyping in Stratifying Patients With Systemic Autoimmune Diseases

The strategy of precision medicine involves patient stratification to improve diagnosis and treatment outcomes. In brief, the therapeutic target is narrowed by stratifying patients within a single disease. These two factors are important for achieving precision medicine: patient stratification and the use of targeted therapies (2). When both factors work, patients are likely to have good outcomes.

To stratify patients, some methods or strategies (e.g., genomic, proteomics, metabolomics) are considered available similar to cancer care. However, acquiring tissue biopsies from patients with autoimmune diseases is logistically more difficult than acquiring samples from patients with cancer. Peripheral immune cell phenotyping, which clarifies the differentiation stage such as naïve or memory T cells, the differences in lineage or functional differences represented by T helper (Th)1 and Th2 cells or Th17 cells, and the activation status or involvement of cellular signaling molecules in the pathological process, is useful for classifying individual patients based on immunological characteristics and can often reflect the pathological condition of involved organs or tissues themselves (32).

We performed peripheral immune cell phenotyping in 16 patients with IgG4-RD (28). Compared with healthy controls (HCs), IgG4-RD showed comparable proportions of Th1 and Th17 cells but higher proportions of Treg and follicular helper T (Tfh) cells. The proportions of class-switched memory B cells, particularly plasmablasts, were higher in IgG4-RD. A histopathological examination revealed marked Tfh cell infiltration, and the increase in Tfh cells in the peripheral blood reflected their degree of infiltration into the tissue. It indicated that peripheral immune cell phenotyping can reveal the immunological status and reflect the immunological condition of the involved lesion (tissue and organ). The abundance of Tfh cells was reported also in another cohort with 15 IgG4-RD patients (33). In this study, there was a correlation between Tfh2 cells, serum IgG4 levels and IL-4 producing plasmablasts. Various other attempts of immune phenotyping in IgG4-RD patients have been performed. In the study with 67 IgG4-RD patients, the frequency of circulating pan-innate lymphoid cells (ILCs) and ILC1s were lower than in HCs, whereas circulating ILC2s were higher in IgG4-RD. Circulating ILC2s correlated positively with CD19^+^ B cells, serum IgG4 and IgE levels (34). In 48 patients with IgG4-RD, circulating CD27^low^CD28^low^CD57^high^ CD4^+^ CTLs were identified as a dominant effector subset. There were prominent infiltration of Granzyme A-expressing CD8^+^ CTLs in involved tissue and clonal expansion of effector/memory CD8^+^ T cells in blood (35). This abundance of CD4^+^ CTLs in blood and tissues were observed also in another cohort with 101 IgG4-RD patients. After clinical remission by B cell-depletion therapy with rituximab, the resolution of these CD4^+^ CTLs were demonstrated (36). The role of immune cells in pathogenesis of IgG4-RD and treatment impact are becoming clearer by immune phenotyping (37).

We also attempted to stratify 143 patients with SLE by immune phenotyping (27). We showed that patients with SLE can be statistically stratified into three subgroups: patients who did not show characteristic features other than a high proportion of plasmablasts, those with a high percentage of Tfh cells, and those with a high percentage of activated and memory Treg cells and a low percentage of naïve Treg cells. Similar attempt in patients' stratification based on immune phenotyping was performed in 105 IgG4-RD patients. In this study, IgG4-RD patients were divided into 3 subgroups by cluster analysis: subgroup 1 with low memory B cells and normal Breg, subgroup 2 with high memory B cells and low Breg, and subgroup 3 with high plasmablasts and low naive B cells. Subgroups 2 and 3 were more likely to be resistant to treatment (38). Patients with a high percentage of Tfh cells were more resistant to treatment with immunosuppressants, in addition to high-dose of glucocorticoids (39). The proportions of CXCR5^+^ CCR7^low^ PD-1^high^ Tfh cells or CXCR5^high^ ICOS^high^ PD-1^high^ Tfh cells were also reported to be associated with disease activity in SLE (40, 41). Not only peripheral immune phenotyping, mass cytometry is identifying responsible cell subsets and markers characteristic of SLE heterogeneity. Transcriptome analysis is discovering molecular networks responsible for disease activity, disease subtype and future relapse. The elucidation of disease heterogeneity in SLE toward further development of precision medicine is becoming clearer by immune cells phenotyping and recent technological advances in single-cell and omics analysis (42).

Peripheral blood lymphocyte phenotyping in 108 patients with RA revealed that the proportions of Tfh, IgD^−^ CD27^−^ double-negative B cells, and plasmacytoid dendritic cells (pDCs) were higher in patients with active RA than in HCs (29). Treatment with TNF-is reduced the proportion of pDCs, while tocilizumab reduced the proportion of double-negative B cells but increased proportions of naïve and activated Treg cells. Notably, the proportion of T follicular helper cells in the peripheral blood was an independent predictor of favorable responses to treatment with abatacept. In rheumatoid arthritis, increasing number of studies based on immune cell phenotyping and other procedures are performed to reveal the genetic, cellular and molecular heterogeneity for further development of precision medicine (43).

We also demonstrated that excessive B-cell differentiation, defined as the proportion of class-switched memory B cells or IgD^−^CD27^−^ B cells among all B cells that was >2 SD higher than the mean in HCs, was associated with treatment resistance in ANCA-associated vasculitis and that rituximab was more effective in patients with circulating B-cell abnormalities (30). Thus, molecular-targeted therapies induced different changes in different immune cell phenotypes, and immune phenotyping might be useful for predicting responses to treatment. As described, recently, there has been much effort toward development of precision medicine in autoimmune diseases around the world.

We next attempted to practice precision medicine for PsA in the real clinical setting as a representative heterogeneous rheumatic disease (autoimmune disease) similar to SLE. However, contrary to SLE, ANCA-associated vasculitis, and IgG4-RD, various types of available molecular-targeting therapies such as bDMARDs and JAK-i are currently under development.

## Precision Medicine Based on Phenotypic Differences in Peripheral T Helper Cells in PsA

Several predictors of treatment response in PsA have been reported (44–50). Biomarkers are also reported predictors (51). However, attempts to establish precision medicine based on these predictors are insufficient. Evidence of the use of precision medicine is lacking. We previously conducted a systematic literature review of PubMed and the Cochrane Library for our literature search from January 2000 to July 2019 (52). However, a quantitative meta-analysis could not be conducted. In PsA, there is insufficient evidence of a quantitative meta-analysis, and our report is currently the only study demonstrating the potential of precision medicine in the real clinical setting.

We reported the potential of precision medicine using bDMARDs based on lymphocyte phenotypes in PsA (31). The first group (strategic bDMARDs) included 26 patients treated with bDMARDs that were selected according to immune cells phenotyping flow cytometry. The second group (standard bDMARDs) included 38 patients who started treatment with a TNF-i that was selected according to the 2015 EULAR or GRAPPA treatment recommendations.

**Figure 1:**
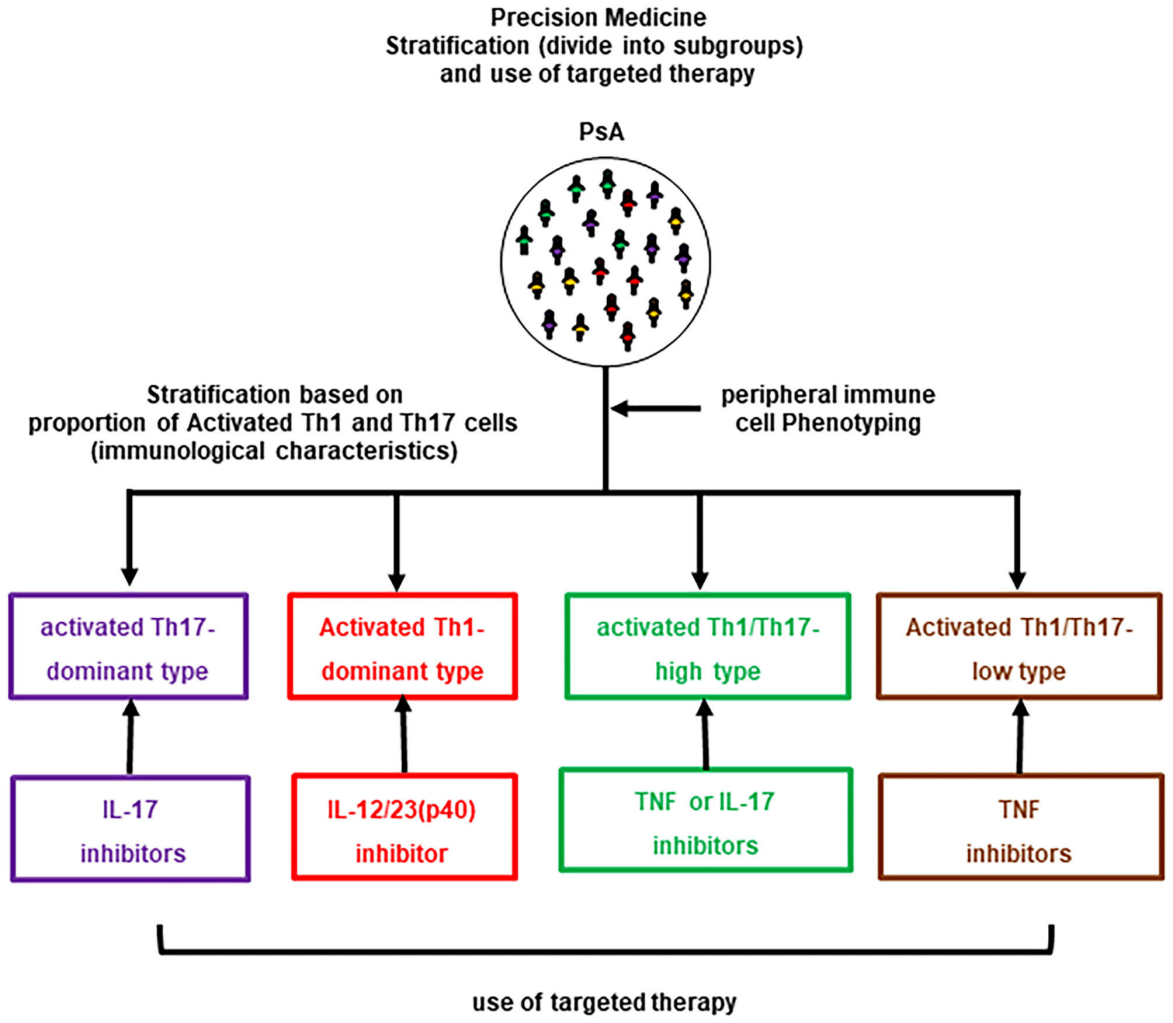
Selective use of bDMARDs in PsA based on the lymphocyte phenotype.

The results revealed that patients with PsA could be classified into four groups based on lymphocyte phenotypes: CD3^+^CD4^+^CXCR3^−^CCR6^+^CD38^+^HLA-DR^+^-activated Th17-dominance, CD3^+^CD4^+^CXCR3^+^CCR6^−^CD38^+^HLA-DR^+^-activated Th1-dominance, activated Th1/Th17-high (hybrid pattern), and activated Th1/Th17-low (normal pattern / comparable with HCs). In the preliminary assessment prior to this study, decreases in activated Th17 cells and improvements in arthritis and skin lesions were observed in activated Th17-dominant patients treated with IL-17-i. Similarly, a decrease in activated Th1 cells and improvements in arthritis and skin lesions were observed in activated Th1-dominant patients treated with IL-12/23(p40)-i, while a decrease in activated Th1/Th17 cells and improvement in arthritis and skin lesions were observed in Th1/Th17-high patients who received TNF-i. Other combinations of treatment and a lymphocyte phenotype resulted in poor or no changes in clinical improvement and/or a lymphocyte phenotype. Therefore, the following treatments were administered to patients according to their lymphocyte phenotype: Ustekinumab (UST) for patients with activated Th1-dominance, IL-17-i for patients with activated Th17-dominance, TNF-i for patients with hybrid pattern and major joint complaints, IL-17-i for patients with a hybrid pattern and major skin complaints, and TNF-i for patients with a normal pattern (Figure 1). Six months after treatment, an intergroup comparison of the efficacy showed that the proportion of patients achieving low disease activity based on Simplified Disease Activity Index (SDAI) was significantly higher in the strategic bDMARDs treatment group (92.3%) than in the standard bDMARDs treatment group (55.2%).

We also assessed the correlation of activated Th1/Th17 cells and clinical severity (SDAI and PASI) in each group, e.g., the association between activated Th17 cells and PASI/SDAI in activated Th17 dominant type. However, there was no significant correlation between activated Th1 or Th17 cells and clinical severity. In this context, it was assumed that the phenotypes of peripheral T helper cells might reflect the latent pathological condition in individual patients rather than reflecting overall PsA disease activity. This study excluded IL-23(p19) and JAK-i since they were recently approved and have just become available in the clinical setting with high efficacy. Based on their mode of action, Il-23(p19)-i is assumed to be more effective in activated Th17 cells-dominant type. Regarding JAK-i, different JAK-is have selectivity for different JAK isoforms and are unlikely to perform equally well for all PsA patients. Further assessment is required to establish precision medicine approach using JAK-i. However, this strategy has not been validated anywhere. Due to the pragmatic reason, we performed CCR6 and CXCR3 staining. It seems to be over simplistic. Other markers, such as CD161 which is enriched on Th17, allow for more accurate immunophenotype. In addition, involvement of many types of immune cells, such as tissue-resident memory T cells, mucosal-associated invariant T cells, ILCs, have recently been reported. More detailed assessment is essential to validate this strategy or evaluate changes in these immune cells including CD8^+^ T cells. There are still limitations, including the fact that the method used in our study, peripheral immune cell phenotyping, is complex and feasible only at limited number of facilities. Peripheral immune cell phenotyping provides a great deal of information, but it has limitations in terms of its versatility. In addition, the advantage of strategic treatment was observed only in a low disease activity achieving rate. The establishment of strategy that leads to more effective control of disease such as achievement of DAPSA-remission, PASI 90 or zero and Minimal Disease Activity is preferable. We are currently investigating whether it can be substituted by measuring serum cytokines. Conversely, single-cell multi-omics analyses of cell surface proteins and gene expression profiles of tissue-infiltrating cells, can enable more detailed and stricter patients' stratification. Such patients-stratification would enable for higher efficacy in molecular-targeted therapy. Therefore, further accumulation of cases, including the detailed assessment of novel biologics or kinase inhibitors, is necessary to establish precision medicine in real world clinical settings.

## Perspective of Further Development of Precision Medicine

There are challenges to the treatment of autoimmune disease using a precision medicine approach vs. utilizing the same approach to treat cancer. First, the development of targeted drugs for autoimmune diseases has historically been slower than that for cancer, and a limited number of treatments are currently available. However, several targeted drugs are currently under development. Second, acquiring tissue biopsies from patients with autoimmune diseases is logistically more difficult than acquiring samples from patients with cancer. In the treatment of malignant tumors, a biopsy is performed to confirm the diagnosis or pathological features and is often readily available for laboratory investigations. This makes it challenging to identify genetic and biochemical differences that contribute to disease in individual patients.

The biopsy-driven observational studies that enrolled RA patients have suggested that certain synovial tissue signatures are associated with treatment response to TNF-i, IL-6 and B-cell depletion therapy (53–57). However, autoimmune diseases such as RA are usually treated without biopsy (joint biopsy). In PsA, contrary to other systemic autoimmune diseases, it may be relatively easier to obtain tissues (skin). The integration of information from tissue biopsies and peripheral immune cell phenotypes or serum cytokine profiles and others may contribute to the further development of novel stratification and more effective treatment strategies in PsA.

Several studies suggest that liquid biopsies are useful to guide therapeutic decisions in cancer (58). In a study using RNA-sequencing data from blood samples, two distinct 23-gene transcriptional signatures to distinguish responders to TNF-i or rituximab were identified (59). In RA, differences in a chromosome conformation signature in blood have been identified as baseline predictive markers of methotrexate treatment (60). In addition to biopsy driven studies, liquid biopsy is also likely to offer important insights into disease pathogenesis and add potential value to precision medicine. If the consistent clinical benefit of treatment strategy based on biopsied-tissue (synovium) information, liquid biopsy and other methods are proven, they will be valuable methods to practice precision medicine in PsA and other systemic autoimmune disease in real world clinical settings.

As mentioned, our study showed changes in phenotypes among patients who achieved a favorable treatment response to bDMARDs. However, evaluations of the association between changes in phenotypes after treatment and treatment responses are insufficient. Moreover, we have not yet sufficiently evaluated temporal factors such as how phenotypes change over time in patients with relapse, whether there are phenotypes that predict treatment resistance already at the time of onset, or whether treatment resistance is acquired during the disease course. Elucidation of this point may provide important insights not only in which b/tsDMARDs should be selected, but also in predicting whether patients need to be treated with b/tsDMARDS or not. Unlike cancer, systemic autoimmune disease has a long (chronic) clinical course. Immunological phenotypes are expected to differ at onset and sometimes thereafter. If immune cell phenotyping can reveal temporal changes in phenotypes, in other words, the immunological natural history of disease, it may contribute to the establishment of precision medicine with the consideration of temporal factors unique to systemic autoimmune diseases with a chronic course.

## Conclusion

Precision medicine has not been sufficiently investigated in real-world clinical settings. However, the dawn of precision medicine has emerged. Hence, we should shed further light on precision medicine in PsA and other autoimmune diseases.

## Author Contributions

IM and YT: substantial contributions to review conception, interpretation of reviewed literature, drafting the article, revising it critically for important intellectual content, and final approval of the version of the article to be published. Both authors have read and agreed to the published version of the manuscript.

## Conflict of Interest

YT has received speaking fees and/or honoraria from Daiichi-Sankyo, Eli Lilly, Novartis, YL Biologics, Bristol-Myers, Eisai, Chugai, Abbvie, Astellas, Pfizer, Sanofi, Asahi-kasei, GSK, Mitsubishi-Tanabe, Gilead, and Janssen; research grants from Abbvie, Mitsubishi-Tanabe, Chugai, Asahi-Kasei, Eisai, Takeda, and Daiichi-Sankyo; and consultant fees from Eli Lilly, Daiichi-Sankyo, Taisho, Ayumi, Sanofi, GSK, and Abbvie. The remaining author declares that the research was conducted in the absence of any commercial or financial relationships that could be construed as a potential conflict of interest.

## Publisher's Note

All claims expressed in this article are solely those of the authors and do not necessarily represent those of their affiliated organizations, or those of the publisher, the editors and the reviewers. Any product that may be evaluated in this article, or claim that may be made by its manufacturer, is not guaranteed or endorsed by the publisher.
